# Notch Dosage*: Jagged1* Haploinsufficiency Is Associated With Reduced Neuronal Division and Disruption of Periglomerular Interneurons in Mice

**DOI:** 10.3389/fcell.2020.00113

**Published:** 2020-02-26

**Authors:** Christopher A. Blackwood, Alessandro Bailetti, Sayan Nandi, Thomas Gridley, Jean M. Hébert

**Affiliations:** ^1^Molecular Neuropsychiatry Research Branch, National Institutes of Health/National Institute on Drug Abuse Intramural Research Program, Baltimore, MD, United States; ^2^Department of Biomedical Sciences, Cornell University, Ithaca, NY, United States; ^3^Departments of Neuroscience and Genetics, Albert Einstein College of Medicine, New York, NY, United States; ^4^Maine Medical Center Research Institute, Scarborough, ME, United States

**Keywords:** *Notch*, neurogenesis, olfactory bulb, interneurons, lateral ganglionic eminence, Alagille syndrome, rostral migratory stream, neural stem/progenitor cells

## Abstract

Neural stem cells in the lateral ganglionic eminence (LGE) generate progenitors that migrate through the rostral migratory stream (RMS) to repopulate olfactory bulb (OB) interneurons, but the regulation of this process is poorly defined. The evolutionarily conserved Notch pathway is essential for neural development and maintenance of neural stem cells. Jagged1, a Notch ligand, is required for stem cell maintenance. In humans, heterozygous mutations in JAGGED1 cause Alagille syndrome, a genetic disorder characterized by complications such as cognitive impairment and reduced number of bile ducts in the liver, suggesting the presence of a JAGGED1 haploinsufficient phenotype. Here, we examine the role of Jagged1 using a conditional loss-of-function allele in the nervous system. We show that heterozygous *Jagged1* mice possess a haploinsufficient phenotype that is associated with a reduction in size of the LGE, a reduced proliferative state, and fewer progenitor cells in the LGE and RMS. Moreover, loss of Jagged1 leads to deficits in periglomerular interneurons in the OB. Our results support a dose-dependent role for Jagged1 in maintaining progenitor division within the LGE and RMS.

## Introduction

The Notch signaling pathway plays multiple roles during the development of the nervous system, including stem cell maintenance, glial differentiation, cell survival, neuronal migration, and neurite formation (Lutolf et al., 2002; Breunig et al., 2007; Hashimoto-Torii et al., 2008). Notch signaling is also important throughout postnatal and adult life. In the subventricular zone (SVZ), Notch signaling is essential for promoting stem cell maintenance and cell division (Nyfeler et al., 2005; Imayoshi et al., 2010; Basak et al., 2012).

In mammals, there are four Notch receptors (Notch1–4) and five ligands [Delta-like 1, 3, and 4, and Jagged1 (Jag1) and 2 (Jag2)] (Kopan and Ilagan, 2009). Binding of a ligand to a Notch receptor leads to a series of cleavage events that results in the release of the intracellular domain (ICD) of Notch. The ICD translocates to the nucleus and forms a transcriptional complex that drives the expression of *Notch* target genes. Within embryonic stem cells, Notch signaling stimulates the expression of transcription factors that inhibit neuronal differentiation, thereby acting to maintain the stem cell population (Ohtsuka et al., 1999; Hatakeyama et al., 2004). Loss-of-function mutations of Jag1 prevent progenitors from generating mature cell types during inner ear development (Hao et al., 2012). Despite these studies, the specific role of Notch signaling on embryonic progenitors that give rise to mature interneurons is comparatively less understood.

Since Notch signaling does not involve a second messenger cascade, it is exquisitely sensitive to the degree of receptor activation (Guruharsha et al., 2012). Heterozygous *Notch* receptor mutations are well known to produce haploinsufficient phenotypes. In *Drosophila*, *Notch* receptor haploinsufficiency results in the classic notched wing phenotype (Morgan, 1917). Haploinsufficient phenotypes associated with mutations in various *Notch* ligands have also been identified. For example, *Delta-like4* haploinsufficiency leads to vascular malformations and embryonic lethality (Gale et al., 2004; Krebs et al., 2004). The importance of *Notch* haploinsufficiency is underscored in studies of Alagille syndrome, which can be caused by heterozygous mutations in either *JAG1* or *NOTCH2* (Li et al., 1997; McCright et al., 2002; McDaniell et al., 2006; Turnpenny and Ellard, 2012; Huppert, 2016; Thakurdas et al., 2016). These patients possess liver, cardiac, and cognitive defects, among others (Alagille et al., 1975; Krantz et al., 1997). Some aspects of these features are seen in mice heterozygous for *Jag1* (Humphreys et al., 2012; Sargin et al., 2013; Thakurdas et al., 2016). In mice, *Jag1* haploinsufficiency is associated with spatial memory impairment (Sargin et al., 2013). Thus, reduction of *Notch* signaling via reduced ligand levels, reduced receptor levels, or both can have effects on organ morphogenesis, development, and adulthood. However, haploinsufficient phenotypes associated with Notch signaling in the brain have not been extensively characterized.

To study the loss-of-function effects of Notch signaling components on brain development, we focused on the role of the Jag1 ligand within the lateral ganglionic eminence (LGE), the precursor to the SVZ among other structures. The postnatal SVZ in mice is essential for the production of mature interneurons within the olfactory bulb (OB) (Doetsch et al., 1999; Lim and Alvarez-Buylla, 2016). *Jag1* has been shown to be essential for maintaining proliferation in the cortical SVZ (Blackwood, 2019) and postnatal SVZ (Nyfeler et al., 2005). Whether Jag1 level is critical in the development of embryonically derived interneurons within the OB remains elusive.

Here we used the *Foxg1*^*Cre*^ driver (Hébert and McConnell, 2000) and a conditional loss-of-function allele of *Jag1* (Kiernan et al., 2006) to disrupt Jag1 function. Homozygous *Jag1* mutants showed a reduced proliferative level within the LGE and rostral migratory stream (RMS). They further displayed diminished numbers of interneuron precursors within the LGE, RMS, and mature interneurons within the OB. Interestingly, *Jag1* homozygous mutant phenotypes were recapitulated in *Jag1* heterozygous mutant mice at varying degrees. Our results demonstrate that the Jag1 signal must be maintained at a critical threshold for proper progenitor division within the LGE.

## Materials and Methods

### Mice

The animals were housed in the AAALAC-accredited East Campus Research Facility and Transgenic Mouse Core Facility in the Veterinary College of Cornell University (CU). All animal procedures were performed in accordance with the guidelines outlined in the National Institutes of Health (NIH) Guide for the Care and Use of Laboratory Animals, eighth Edition. The study protocol animals were approved by CU’s Animal Care and Use Committee (IACUC; #01-75). *Jag1*^*fl/fl*^ (Kiernan et al., 2006) and *Foxg1*^*Cre*^ (Hébert and McConnell, 2000). Mice were genotyped by PCR (forward: 5′-TCAGGCATGATAAACCCTAGC-3′ and reverse: 3′-CTACATACAGCATCTACATGC-5′) primers. Mice were maintained on a mixed 129Sv/C57BL/6 background and housed on a reverse light–dark cycle. Food and water were continuously available. Male and female mice mated overnight. The following morning females were separated and checked for a vaginal plug. Pregnant mice were euthanized using CO_2_ asphyxiation followed by cervical dislocation consistent with the recommendations of the Panel on Euthanasia of the American Veterinary Medical Association and the CU IACUC.

### *In situ* Hybridization

*In situ* hybridization was performed as previously described using sagittal or coronal brain 20 μm sections (Rodriguez et al., 2008). Briefly, embryonic day 17.5 (E17.5; E0.5 was defined as the first detection of the vaginal plug) embryos were dissected and decapitated. Heads were embedded in O.C.T. compound (Tissue-Tek, 25608-930) and fresh-frozen in liquid nitrogen-cooled isopentane. Previously published probe sequences were used for *Notch1-3* (Rodriguez et al., 2008), *Dlx2*, *Jag1*, and *GluR1* (Paek et al., 2009; Williams et al., 2011). Additionally, *Delta1*, *Jag2*, *EGFR*, *TH*, *Mash1*, and *Sox2* probes were derived from the Brain Molecular Anatomy Project (BMAP) or National Institute of Aging (NIA) 15 k or 7.4 k clone sets. Sections were incubated with RNA probes for 48 h at 67°C and washed with 5× SSC followed by 0.2× SSC. Afterward, slides were cooled to room temperature (RT) and blocked with TNB (0.1 M Tris–HCl pH 7.5, 0.15 M NaCl, 0.5% blocking reagent) (Perkin Elmer). Anti-digoxigenin-alkaline phosphatase antibody was applied to slides (1:3000, Roche) overnight at 4°C. Slides were washed with B1 Tween buffer (100 mM Tris pH 7.4, 150 mM NaCl, Tween 0.05%), followed by B3 buffer (100 mM Tris pH 9.5, 50 mM MgCl, 100 mM NaCl) and reacted in NBT/BCIP (Promega).

### Double-Labeled *in situ* Hybridization

Embryonic day 17.5 brains were embedded in O.C.T. compound (Tissue-Tek, 25608-930) and 10 μm fresh-frozen cryosections were fixed in 4% paraformaldehyde, washed in phosphate-buffered saline, and acetylated with 0.25% acetic anhydride in 0.1 M triethanolamine, pH 8.0. Slides were blocked with blocking reagent (Roche, 11096176001) for 2 h according to the manufacturer’s protocol. Slides were then hybridized with both digoxigenin-labeled and biotin-labeled antisense RNA probes for 48 h at 70°C and washed with 5× SSC followed by 0.2× SSC. Afterward, slides were cooled to RT and blocked with TNB (0.1 M Tris–HCl pH 7.5, 0.15 M NaCl, 0.5% blocking reagent) (Perkin Elmer). Anti-digoxigenin-alkaline phosphatase antibody was applied to slides (1:3000, Roche) overnight at 4°C. Slides were washed with B1 Tween buffer (100 mM Tris pH 7.4, 150 mM NaCl, Tween 0.05%), followed by B3 buffer (100 mM Tris pH 9.5, 50 mM MgCl, 100 mM NaCl). Slides were incubated with 200 μl of SA-HRP (1:100; Abcam) in TNB for 30 min at RT. Slides were washed in B1 buffer with Tween (0.5%), and incubated in 200 μl of biotinyl tyramide (1:50, Perkin Elmer) solution of for 10 min. Subsequently, slides were incubated in 200 μl of SA-Alexa Fluor 488 (1:200) in TNB for 30 min at RT, washed in B1, and then washed in Fast Red Buffer. Fast Red TR/HNPP (HNPP Fluorescent Detection Set; Roche) was applied according to the manufacturer’s instructions.

### Western Blotting

Western blot was performed as previously described (Blackwood et al., 2018). In brief, the area surrounding the dLGE at E17.5 was dissected and homogenized in RIPA buffer (10 mM Tris 7.4, 150 mM NaCl, 0.1% SDS, 1% Triton X-100, 1% deoxycholate, and 5 mM EDTA) containing protease inhibitors (Roche), and then clarified by centrifugation (4°C, 10 min, 15,000 × *g*). Western blotting was performed with 10 μg of lysate and detection of protein was accomplished with primary antibodies directed against Jag1 (1:200, Santa Cruz, SC-6011) or alpha tubulin (1:2000, Sigma, SAB3501072). The secondary antibodies used were rabbit anti-goat-HRP (1:500, ThermoFisher Scientific; G-21234) and goat anti-rabbit-HRP (1:500, Sigma, 12-348).

### Histology and Immunohistochemistry

For histology, E17.5 embryos were fresh-frozen in O.C.T. compound (Tissue-Tek, 25608-930) using liquid nitrogen-cooled-isopentane solution. Twenty micrometer-thick sections were obtained and fixed in 4% phosphate-buffered formaldehyde (pH 7.4). For Nissl staining, slides were rinsed three times with distilled water, treated with 0.2% acetate buffer (pH 4.0; 2 min), stained with 0.1% cresyl violet (5–10 min), rinsed with water, and mounted in 70% glycerol. For immunohistochemistry (IHC), antigen retrieval was first performed with citrate buffer [10 mM sodium citrate, pH 6.0; 2 min, 70% power (microwave) followed by 8 min, 20% power]. Ki67 (1:100, NovoCastra, NCL-Ki67p, rabbit) and anti-phospho-histone H3 (p-HH3, 1:200, Millipore, 06570, rabbit) antigen reactivity was detected using an Alexa Fluor 568 secondary antibody (Thermo-Fisher, A-11036). For Proliferating Cell Nuclear Antigen (PCNA, 1:1000, Abcam, ab18197) detection slides were incubated overnight with antibody and then allowed to react with biotinylated goat-anti rabbit secondary (1:500, SC-2040) for 1 h at RT followed by Streptavidin-HRP (1:500, ThermoFisher Scientific, D22187). Reactivity was observed using the 3,3′-diaminobenzidine (DAB) kit (ThermoFisher Scientific, D22187) according to the manufacturer’s protocol. Images were captured using a Zeiss Axioskop2 Plus.

### Neurosphere Assay

Neurospheres (NS) were generated from the dorsal LGE (dLGE) region of individual E17.5 embryos using a similar method previously described in Blackwood (2019), but independent cultures were used to re-examine the *in vitro* phenotype. The two hemispheres were separated and the area around the lateral ventricles was carefully isolated. The tissue was then digested with 0.25% trypsin-EDTA (37°C, 15 min). The pellet was re-suspended in Hank’s Buffered Salt Solution (HBSS; ThermoFisher Scientific; 14185-052) (37°C, 5 min) and centrifuged (300 × *g*, 3 min). This step was repeated twice to remove any residual trypsin-EDTA and the pellet was re-suspended in 4 ml of HBSS containing 3 mg/ml BSA. The cell pellet was successively triturated with an 18-gauge needle (10 times), a 21-gauge needle (10 times), and 23-gauge needle (5 times) until the suspension appeared uniform. Next, cells were re-suspended in 5 ml of complete media containing Dulbecco’s Modified Eagle Medium/Nutrient Mixture F12 (DMEM/F12) (ThermoFisher Scientific; 11320-033) supplemented with 10% fetal bovine serum containing 20 ng/ml of recombinant human (rh)EGF (ThermoFisher Scientific; PHG0311) and plated at 250 μl with 10^4^ cells in each well in a 48-well plate (Corning; 3538). An additional 100 μl of complete media containing 20 ng/ml rhEGF was added every third day.

### TUNEL Assay

Terminal deoxynucleotidyl transferase dUTP nick end labeling (TUNEL) reactions were carried out on 12–16 μm fresh frozen sections following the manufacturer’s protocol (Roche; 11684795910). Quantification of the TUNEL was performed by counting the total number of apoptotic cells per section in the region of the dLGE, RMS, or OB.

### Data Acquisition and Statistical Analysis

Image importation and quantitation were carried out using ImageJ software. A standardized region of interest was selected for all images and matching littermate sections were processed using the same threshold value. The image was further processed using the binary watershed function to better distinguish individual cells, and the number of positive signal was determined using the particle analyzer function. After watershed processing, a single positive signal was defined as a cell. Percentages in Figure 4 were determined by counting the number of positive signal/cells over the total number of DAPI cells. Data were analyzed using Prism v8.3.0 (GraphPad Software, San Diego, CA, United States) by performing Student’s *t*-test (Supplementary Figures S1, S2) or one-way analysis of variance (ANOVA) (Figures 3–6 and Supplementary Figure S3). Data with statistically significant (*p* < 0.05) *p*-values were further analyzed by Fisher’s PLSD *post hoc* test to perform multiple comparisons between groups (control, heterozygous, mutant) using StatView Version 4.0 (SAS, Cary, NC, United States). The *p*-values from the statistical analyses are provided in Tables 1, 2 for all experiments. The null hypothesis was rejected at *p* < 0.05. Littermate controls were used for quantification. A minimal of three independent embryos was used for quantification of *in situ* hybridizations, IHCs, histology, and neurosphere experiments. The number of sections or pictures used per animal is listed in figure legends. Quantitation of Nissl and *Dlx2* was performed by calculating the ratio of the lengths of the LGE vs. cortex. The segments used to calculate the length of the LGE is defined by drawing a line from the dLGE to the vLGE. The segment used to calculate the length of the cortex is defined by drawing a straight line from the apex of the posterior cortex to the anterior prefrontal cortex. An example of the segments is illustrated in Supplementary Figure S1D. The mean values for LGE, cortex lengths, and ratio are listed in Table 3.

**TABLE 1:** Summary of one-way ANOVA results.

Experiment	Region	*F*-score	*p*-value	*R*^2^	Figures
Western blot	LGE	*F*(2,3) = 53.1	0.0046	0.973	3I
Nissl	LGE	*F*(2,12) = 4.86	0.0285	0.447	3J
*Dlx2*	LGE	*F*(2,12) = 5.71	0.0181	0.488	3K
p-HH3	dLGE	*F*(2,12) = 43.0	0.0001	0.878	4N
Ki67	dLGE	*F*(2,9) = 30.8	0.0001	0.872	4O
PCNA	dLGE	*F*(2,6) = 15.3	0.0044	0.836	4P
*EGFR*	dLGE	*F*(2,7) = 12.6	0.0047	0.783	4Q
NS	dLGE	*F*(2,9) = 63.1	0.0001	0.933	4R
TUNEL	dLGE	*F*(2,15) = 0.469	0.6344	0.0589	4S
PCNA	RMS	*F*(2,9) = 318	0.0001	0.986	5N
Ki67	RMS	*F*(2,12) = 72.3	0.0001	0.923	5O
*MASH1*	RMS	*F*(2,9) = 960	0.0001	0.995	5P
TUNEL	RMS	*F*(2,15) = 0.0168	0.9833	0.00224	5Q
PCNA	OB	*F*(2,12) = 58.0	0.0001	0.906	6K
*TH*	OB (PG)	*F*(5,25) = 29.2	0.0001	0.854	6L
*GluR1*	OB (PG)	*F*(2,13) = 78.2	0.0001	0.923	6M
*TH*-interneurons vs. PCNA cells	OB (mn)	*F*(2,11) = 0.232	0.7967	0.0405	6N
*TH*-interneurons vs. PCNA cells	OB (PG)	*F*(2,11) = 2.571	0.1213	0.319	6O
*GluR1-*interneurons vs. PCNA cells	OB (PG)	*F*(2,11) = 8.883	0.0051	0.618	6P
TUNEL	OB	*F*(2,9) = 0.143	0.8688	0.0318	S3

**TABLE 2:** Summary of *p*-values from the *post hoc* test or Student’s *t*-test.

Experiment	Region	c vs. h	c vs. m	m vs. h	Pos. Cre vs. Neg. Cre	Figures
Western blot	LGE	*p* = 0.0443	*p* = 0.0035	*p* = 0.0479	–	3I
Nissl	LGE	*p* = 0.0087	*p* = 0.0010	*p* = 0.0069	–	3J
*Dlx2*	LGE	*p* = 0.0310	*p* = 0.0463	*p* = 0.0278	–	3K
*Dlx2*	LGE	–	–	–	*p* = 0.1450	S1
p-HH3	dLGE	*p* < 0.0001	*p* < 0.0001	*p* = 0.4082	–	4N
Ki67	dLGE	*p* = 0.0017	*p* = 0.0005	*p* = 0.1623	–	4O
PCNA	dLGE	*p* = 0.0234	*p* = 0.0135	*p* = 0.0139	–	4P
*EGFR*	dLGE	*p* = 0.0261	*p* = 0.0092	*p* = 0.0799	–	4Q
NS	dLGE	*p* = 0.0002	*p* < 0.0001	*p* = 0.0118	–	4R
NS	dLGE	–	–	–	*p* = 0.5826	S2
TUNEL	dLGE	*p* = 0.8520	*p* = 0.3944	*p* = 0.4791	–	4S
PCNA	RMS	*p* < 0.0001	*p* < 0.0001	*p* = 0.0002	–	5N
Ki67	RMS	*p* < 0.0001	*p* < 0.0001	*p* = 0.0012	–	5O
*MASH1*	RMS	*p* < 0.0001	*p* < 0.0001	*p* = 0.0109	–	5P
TUNEL	RMS	*p* = 0.8790	*p* > 0.9999	*p* = 0.8790	–	5Q
PCNA	OB	*p* = 0.0003	*p* < 0.0001	*p* < 0.0001	–	6K
*TH*	OB (PG)	*p* < 0.0001	*p* < 0.0001	*p* < 0.0001	–	6L
*TH*	OB (mn)	*p* = 0.0151	*p* = 0.0011	*p* = 0.0213	–	6L
*GluR1*	OB (PG)	*p* < 0.0001	*p* < 0.0001	*p* = 0.0001	–	6M
*TH*-interneurons vs. PCNA cells	OB (mn)	*p* = 0.7713	*p* = 0.7003	*p* = 0.5413	–	6N
*TH*-interneurons vs. PCNA cells	OB (PG)	*p* = 0.0950	*p* = 0.0869	*p* = 0.3601	–	6O
*GluR1-*interneurons vs. PCNA cells	OB (PG)	*p* = 0.0001	*p* = 0.0117	*p* = 0.0766	–	6P
TUNEL	OB	*p* > 0.05	*p* > 0.05	*p* > 0.05	–	S3

**TABLE 3:** Summary of mean values for LGE and cortex lengths.

Experiment	Genotype	Abbreviation	Average length of LGE ± SEM (μm)	Average length of cortex ± SEM (μm)	Ratio
Nissl	*Foxg1*^+/+^; *Jag1*^*fl/+*^	c	3260 ± 500	5732 ± 236	0.548 ± 0.142
Nissl	*Foxg1*^*Cre/+*^; *Jag1*^*fl/+*^	h	1290 ± 132	5696 ± 187	0.229 ± 0.028
Nissl	*Foxg1*^*Cre/+*^; *Jag1*^*fl/f/*^	m	1826 ± 490	5694 ± 207	0.323 ± 0.020
*Dlx2*	*Foxg1*^+/+^; *Jag1*^*fl/+*^	c	3260 ± 560	6332 ± 406	0.524 ± 0.088
*Dlx2*	*Foxg1*^*Cre/+*^; *Jag1*^*fl/+*^	h	990 ± 240	6294 ± 271	0.158 ± 0.005
*Dlx2*	*Foxg1*^*Cre/+*^; *Jag1*^*fl/f/*^	m	1366 ± 950	6306 ± 288	0.217 ± 0.013
*Dlx2*	*Foxg1*^+/+^	–	2234 ± 137	7536 ± 273	0.296 ± 0.013
*Dlx2*	*Foxg1*^*Cre/+*^	–	2367 ± 102	7786 ± 318	0.306 ± 0.017

## Results

### *Jag1* Expression Coincides With Neurogenesis in the dLGE

**FIGURE 1:**
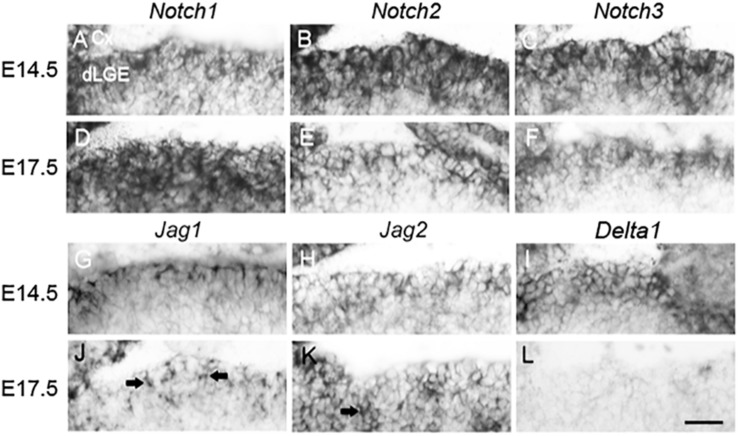
*Notch* receptor and ligand expression patterns in the dorsal lateral ganglionic eminence. **(A–L)** RNA *in situ* hybridization of sagittal brain sections of *Notch* receptors and *Notch* ligands. At E14.5 and E17.5 the expression of **(A,D)** *Notch1*, **(B,E)** *Notch2*, and **(C,F)** *Notch3* probes was strongly detected. At E14.5, positive signal was also detected for **(G)** *Jag1*, **(H)** *Jag2*, and **(I)** *Delta1* probes. At E17.5, **(J)** *Jag1* and **(K)** *Jag2* probes were expressed (arrows indicate positive expression). Conversely, at E17.5, **(L)** *Delta1* probe shows weak or no expression. Scale bar = 30 μm. Cx, cortex; dLGE, dorsal lateral ganglionic eminence.

**FIGURE 2:**
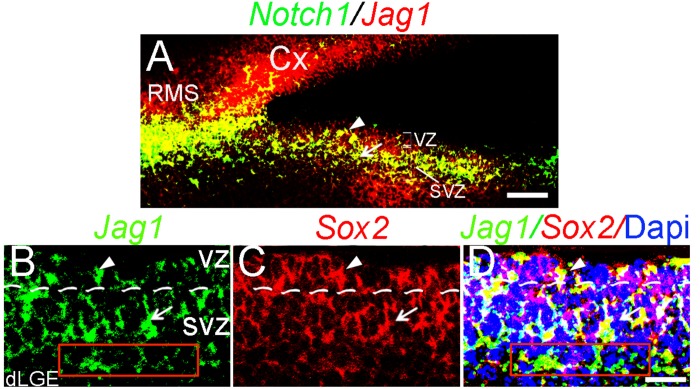
*Jag1* is expressed in VZ, SVZ, and rostral migratory stream. **(A–D)** *In situ* hybridization expression of *Jag1*, *Notch1*, and *Sox2* mRNA probes on adjacent sagittal brain sections at E17.5. **(A)** Merged images of *Notch1* and *Jagged1* show coexpression in the RMS, VZ, and SVZ. Scale bar = 100 μm. **(B)** Expression of *Jag1* probe (green). **(C)** Expression of *Sox2* probe (red). **(D)** Overlay (yellow) of *Jag1* and *Sox2* expression in the VZ and SVZ counter-stained with DAPI (blue). The VZ (∼1–3 cells from the neuroepithelial surface) and SVZ (∼4–8 cells from the neuroepithelial surface) were measured using DAPI. Scale bar = 30 μm. Arrowheads indicate VZ; arrows indicate SVZ. Cx, cortex; RMS, rostral migratory stream; VZ, ventricular zone; SVZ, subventricular zone.

The dLGE contains multipotent neural stem/progenitor cells that are regulated by *Notch* Signaling and that generate bulbar interneurons (Androutsellis-Theotokis et al., 2006; Imayoshi et al., 2010; Qin et al., 2017). A vast majority of bulbar periglomerular interneurons are generated from the LGE beginning at embryonic day 14.5 (E14.5) and continuing through development (Luskin, 1993; Tucker et al., 2006; Batista-Brito et al., 2008; Humphreys et al., 2012). At E14.5 and E17.5, we examined the expression pattern of *Notch* receptors and a subset of *Notch* ligands in the dLGE. Three of the four mammalian *Notch* receptors were detected at both E14.5 (Figures 1A–C) and E17.5 (Figures 1D–F). Similarly, at E14.5 the expression of *Jag1*, *Jag2*, and *Delta1* was also observed (Figures 1G–I). Furthermore, *Jag1* (Figures 1J, 2A) and *Jag2* (Figure 1K) were also detectable at E17.5; however, weak to no signal was found for *Delta1* (Figure 1L). Since the appearance of *Jag1* at E17.5 coincided with the peak of OB neurogenesis (∼E18.0) (Hinds, 1968), we further examined the role of *Jag1* during late embryonic development (E17.5).

**FIGURE 3:**
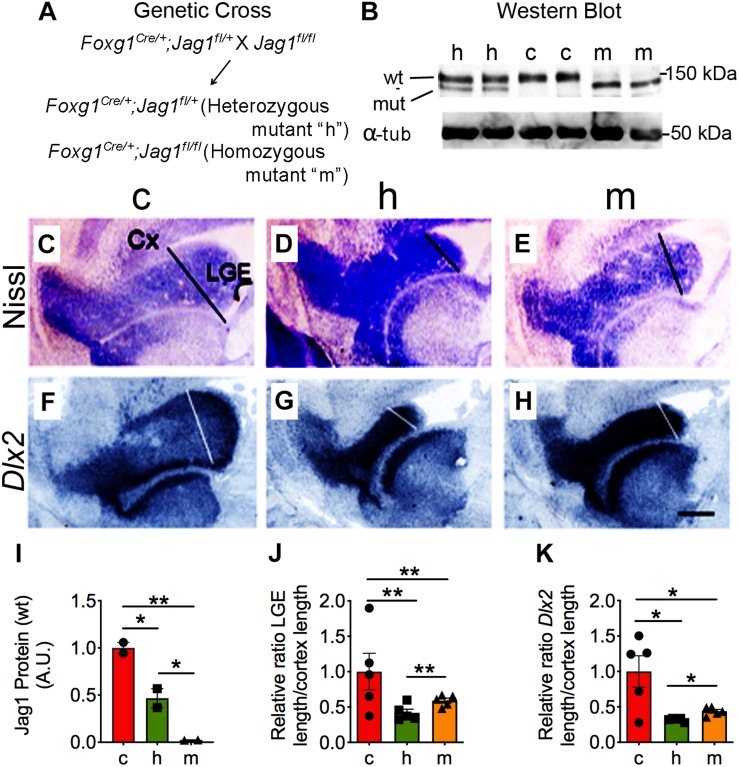
Haploinsufficient *Jag1* signaling shows reduced size of the LGE. **(A)** Schematic for the generation of *Jag1* heterozygous (h) and homozygous (m) mutants. **(B,I)** Tissue from dLGE at E17.5 used in western blot shows substantial reduction in full-length (wt) Jag1 protein (150 kDa) expression in heterozygous and homozygous brains (*n* = 2). Top band represents control (wt) and lower band mutant (mut) Jag1 protein. **(C–E,J)** The length of the Nissl staining in the LGE was significantly reduced in heterozygous and homozygous mice (c, *n* = 5; h, *n* = 5; m, *n* = 5). **(F–H,K)** The length of the expression of *Dlx2 in situ* probe was significantly reduced in both mutants (c, *n* = 5; h, *n* = 5; m, *n* = 5). Length for Nissl and *Dlx2* LGE regions was determined by drawing a line from the point where the cortex meets the LGE to the point where the LGE connects with the ventral ventricle. All graphs show average ± SEM. Key to statistics: *p < 0.05; **p < 0.01, respectively, in comparison to control or heterozygous. Five independent embryos (*n* = 5) were counted and one section was selected to quantify for each genotype in Nissl and Dlx2 experiments. Scale bar = 300 μm. Cx, cortex; LGE, lateral ganglionic eminence; wt, wild-type; A.U., arbitrary units; NS, not significant.

**FIGURE 4:**
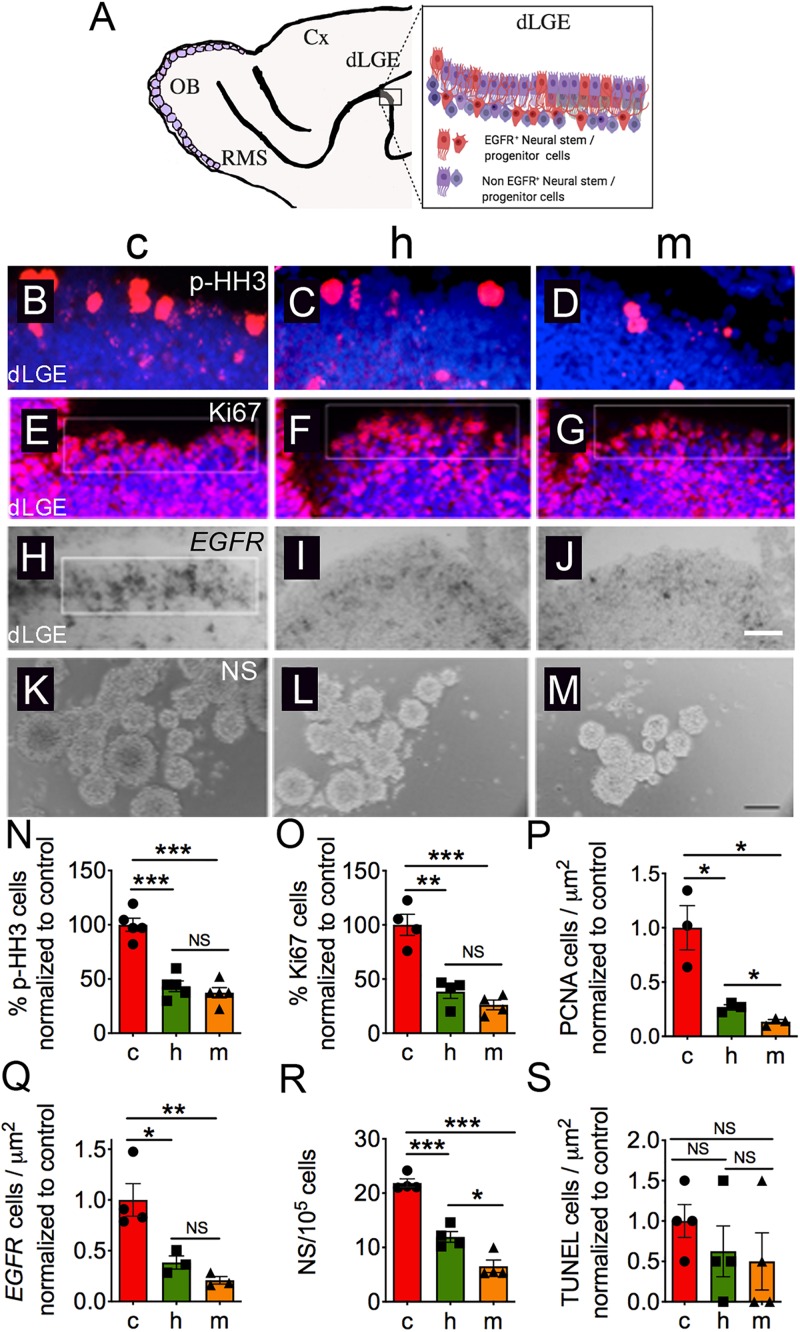
Haploinsufficient *Jag1* signaling strongly attenuates neuronal division in the dLGE. **(A)** Schematic cartoon of the dLGE. E17.5 sagittal brain sections show reduced immunostaining of **(B–D,N)** p-HH3 (c, *n* = 5; h, *n* = 5; m, *n* = 5) and **(E–G,O)** Ki67 (c, *n* = 4; h, *n* = 4; m, *n* = 4) antibodies in the heterozygous and homozygous mice in the dorsal LGE. Sections were counterstained with DAPI (blue). Quantitations were performed for p-HH3^+^ and Ki67^+^ cells (white box; area = ∼82,000 μm^2^) by counting the number of positive cells over the total number of DAPI^+^ cells. Similarly, **(P)** quantification of PCNA shows a significant reduction in *Jag1* mutants (c, *n* = 3; h, *n* = 3; m, *n* = 3). **(H–J,Q)** Reduction in *EGFR*^+^ cells in the heterozygous (*n* = 3) and homozygous mutants (*n* = 3) in the dorsal LGE (c, *n* = 4). Quantitation was performed by counting the number of *EGFR*^+^ cells in the boxed region (area = ∼74,880 μm^2^) of each image, determining the number of positive cells micron. **(K–M,R)** Cells isolated from the dLGE show fewer numbers of neurospheres (c, *n* = 7; h, *n* = 7; m, *n* = 7; seven randomly selected pictures were used to quantify per genotype from four independent embryos). Scale bar = 100 μm. Neurosphere quantitation was performed at seven DIV by counting the total number of neurospheres at 25× magnification to determine the number of NS per 10^5^ cells. **(S)** Quantification of cell death as determined by TUNEL in the LGE showed no significant differences between mice (c, *n* = 6; h, *n* = 6; m, *n* = 6). All graphs show average ± SEM. Key to statistics: *p < 0.05; **p < 0.01; ***p < 0.001, respectively, in comparison to control or heterozygous; NS, not significant. A minimal of three independent embryos was counted and one to four sections were selected from each genotype. Scale bar = 100 μm for panels **(B–J)**. OB, olfactory bulb; RMS, rostral migratory stream; Cx, cortex; dLGE, dorsal lateral ganglionic eminence; NS, neurospheres.

**FIGURE 5:**
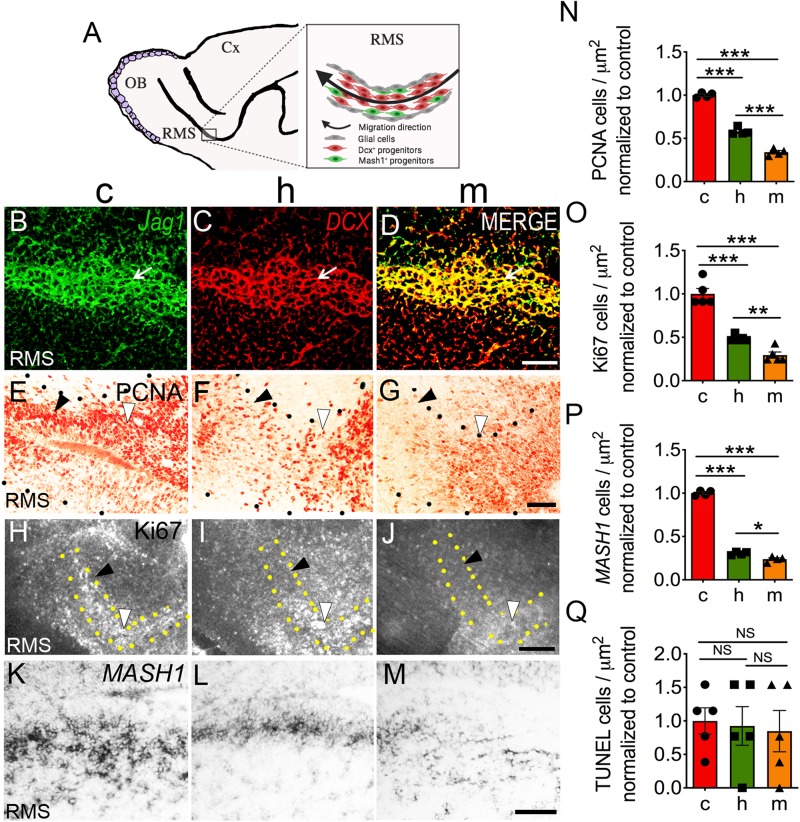
Haploinsufficient in *Jag1* causes defects in the rostral migratory stream. **(A)** Schematic illustration of the RMS. Double-labeled *in situ* hybridization on control brain sections using probes for **(B)** *Jag1* (green) and **(C)** *DCX* (red) shows **(D)** coexpression (yellow) in the RMS (white arrows). Scale bar = 100 μm. Immunohistochemistry using **(E–G)** anti-PCNA and **(H–J)** anti-Ki67 antibodies shows reduction in PCNA (c, *n* = 4; h, *n* = 4; m, *n* = 4) and Ki67 (c, *n* = 5; h, *n* = 5; m, *n* = 5) signals in **(F,I)** heterozygous and **(G,J)** homozygous mutants, respectively (black arrowheads). Scale bar = 50 μm (PCNA) and 250 μm (Ki67). **(K–M)** The number of *Mash1*^+^ cells decline in the heterozygous and homozygous mice (c, *n* = 4; h, *n* = 4; m, *n* = 4). Scale bar = 50 μm. Graphs **(N–P)** display the quantification of PCNA, Ki67, and *Mash1*. **(Q)** Total count of apoptotic cells using TUNEL shows no changes in cells death (c, *n* = 5; h, *n* = 5; m, *n* = 5). All images are sagittal brain sections at E17.5. White arrowheads indicate the elbow of the RMS. All graphs show average ± SEM. Key to statistics: *p < 0.05; **p < 0.01; ***p < 0.001, respectively, in comparison to control or heterozygous; NS, not significant. A minimal of four independent embryos was counted for each genotype and one to two sections were selected from each genotype. RMS, rostral migratory stream; Cx, cortex.

**FIGURE 6:**
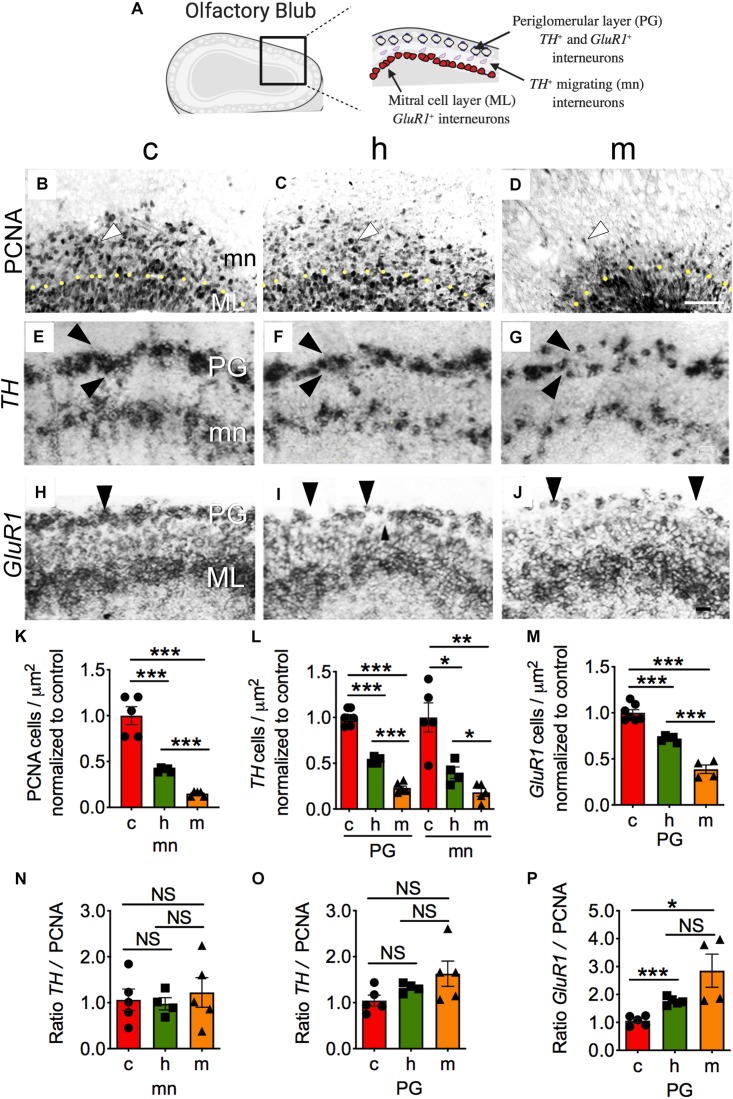
Loss of *Jag1* shows defects in periglomerular interneurons. **(A)** Schematic cartoon of the OB. **(B–D,K)** Immunolabeling with anti-PCNA antibody on sagittal brain sections shows reduction of PCNA^+^ cells (arrowheads) outside the ML (c, *n* = 5; h, *n* = 5; m, *n* = 5). Scale bar = 100 μm. PCNA quantification was performed by counting the number of positive cells per micron outside the ML. RNA *in situ* hybridization using **(E–G,L)** *TH* probe shows decreased signal in the PG (c, *n* = 7; h, *n* = 4; m, *n* = 6) and mn (c, *n* = 5; h, *n* = 4; m, *n* = 5) regions. **(H–J,M)** The number of *GluR1*^+^ cells was reduce (c, *n* = 7; h, *n* = 5; m, *n* = 4) in the PG layer (arrowheads); Scale bar = 30 μm. **(N)** Calculation of bulbar *TH*^+^ interneurons (mn) vs. PCNA cells (mn) ratio displays no substantial differences. **(O)** Normalized ratio of bulbar *TH*^+^ interneurons (PG) vs. PCNA cells (mn) shows no significant changes. **(P)** Normalized ratio of *GluR1*^+^ interneurons (PG) vs. PCNA cells (mn) shows significant increases in heterozygous and homozygous mice. *TH* or *GluR1* counting was performed using a straight line through the region of interest and counting the number of cells per micron. All sections are coronal. All graphs show average ± SEM. Key to statistics: *p < 0.05; **p < 0.01; ***p < 0.001, respectively, in comparison to control or heterozygous; NS, not significant. A minimal of four independent embryos was counted for each genotype and one to three sections were selected from each embryo. PG, periglomerular layer; mn, migrating neurons; ML, mitral cell layer.

### *Jagged1* Is Expressed in at Least a Subset of Neural Stem/Progenitor Cells in the dLGE

*SRY* (*sex determining region Y*)*-box 2^+^* (*Sox2*) -positive neural stem/progenitor cells have been shown to express *Jag1* in the inner ear (Kiernan et al., 2006). We examined whether *Sox2* and *Jag1* are coexpressed in the proliferative zone of the dLGE. Using *in situ* hybridization, we found that the expression of *Jag1* (Figure 2B) appears to coincide with the expression pattern of *Sox2* (Figure 2C) in the VZ and SVZ (Figure 2D). We also detected the expression of *Jag1* in the deep layer of the SVZ (Figures 2B,D; red boxes).

### Haploinsufficient *Jag1* Gene Results in Reduced LGE Size

Null mutations of *Jag1* are embryonic lethal at E10 (Xue et al., 1999). We crossed a conditional *Jag1* allele (*Jag1^*fl*/f*l*^*) (Kiernan et al., 2006) with the *Foxg1*^*Cre*^ driver (Hébert and McConnell, 2000) to remove *Jag1* in telencephalic precursor cells that give rise to the LGE and RMS. *Foxg1*^*Cre*^ driver recombines floxed alleles in telencephalic precursor cells by E9.0 (Hébert and McConnell, 2000). In the presence of Cre, a truncated Jag1 protein is produced. A significant reduction in the full-length Jag1 protein (150 kDa) was found in the heterozygous [−54.27%; (2336 ± 501) A.U.] and homozygous [−97.4%; (126 ± 6.5) A.U.] mice relative to control [(5000 ± 291) A.U.] mice (Figures 3B,I). Furthermore, there was also a substantial difference between the heterozygous and homozygous mutant mice (Figure 3I). This data indicate that, as previously shown by our lab to determine for conditional deletion of *Fibroblast growth receptors* (*Fgfr1*^*f/f*^ and *Fgfr2*^*f/f*^) and *Fibroblast growth factor substrate 2*α (*Frs2*^*f/f*^) floxed alleles (Hébert and McConnell, 2000; Paek et al., 2009; Nandi et al., 2017), the *Foxg1*^*Cre*^ line efficiently deletes *Jag1*^*f/f*^ allele early in the ventral telencephalon. In the analyses described below, “control” refers to either *Jag1* floxed heterozygous or homozygous littermates that are *Foxg1*^*Cre*^-negative.

To determine if and how reduced Jag1 protein affects the LGE, we calculated the length of the LGE, represented as a ratio of the LGE to cortex (for example see Supplementary Figure S1D). The ratio of the lengths is reduced in heterozygous [−58.2%; (0.229 ± 0.028)] and homozygous [−40.0%; (0.323 ± 0.020)] mice compared to control (0.548 ± 0.142) mice (Figures 3C–E,J and Table 3).

*Dlx2* is expressed in neural precursors within the LGE and has been shown to be essential for the generation of bulbar interneurons (Bulfone et al., 1998; Kohwi et al., 2005; Brill et al., 2008; Haba et al., 2009). We also used the expression of *Dlx2*, as a marker, to measure the length of the LGE. The ratio of *Dlx2* length was significantly reduced in both heterozygous [−69.8%; (0.158 ± 0.005)] and homozygous [−58.6%; (0.217 ± 0.013)] mice compared to controls (0.524 ± 0.088) (Figures 3F–H,K and Table 3), which is consistent with the overall reduction in LGE observed with Nissl staining (Figures 3C–E,J). We also observed significant differences in the size of the LGE between the heterozygous and homozygous mice (Figures 3J,K). To confirm that the reduced LGE in *Jag1* mutants was not due to the *Foxg1*^*Cre/+*^ driver, we also determined the ratio of *Dlx2* in these mice. No significant changes in the expression of *Dlx2* were observed in the *Foxg1*^*Cre/+*^ (0.306 ± 0.017) compared to *Foxg1*^+/+^ (0.296 ± 0.013) mice (Supplementary Figures S1A–C and Table 3). Together, these data suggest that deletion of one or two alleles of *Jag1* leads to a reduced length of the LGE.

### Haploinsufficient *Jag1* Mice Strongly Attenuates Neuronal Division in the dLGE

Figure 4A shows a cartoon schematic of the dLGE region. We next sought to identify factors that could explain the reduced size in the LGE. We tested whether the loss of *Jag1* affects cell division in the dLGE. To achieve this goal, we used antibodies against p-HH3 (Hendzel et al., 1997) and Ki67 (Gerlach et al., 1997) to determine the fraction of cells undergoing mitosis and that are proliferative, respectively. The number of cells with p-HH3 expression was significantly reduced in both heterozygous [−56.5%; (9.7 ± 1.1%) cells/DAPI] and homozygous [−62.7%; (8.3 ± 1.0%) cells/DAPI] mice relative to control [(22.3 ± 1.3%) cells/DAPI] mice (Figures 4B–D,N). Moreover, similar reductions were also observed in the number of Ki67 cells in heterozygous [−61.7%; (28.8 ± 4.6%) cells/DAPI] and homozygous [−73.8%; (19.7 ± 3.3%) cells/DAPI] mice in comparison to control [(75.2 ± 7.3%) cells/DAPI] (Figures 4E–G,O). Furthermore, using an antibody against PCNA, which marks dividing cells (Bravo, 1986), we found a significant reduction in heterozygous [−73.3%; (1.20 × 10^–2^ ± 1.11 × 10^–3^) cells/μm^2^] and homozygous [−86.5%; (6.08 × 10^–3^ ± 8.90 × 10^–4^) cells/μm^2^] mice compared to control mice (4.52 × 10^–2^ ± 9.22 × 10^–3^) cells/μm^2^ (Figure 4P). Quantification of the PCNA^+^ cells also showed a substantial difference between the heterozygous and mutant mice (Figure 4P). Due to the reduced proliferative levels in the dLGE of mutants, we asked whether the loss of *Jag1* affects neural stem/progenitor cell numbers. We used a probe for *EGFR* that labels neural stem/progenitor cells (Ciccolini et al., 2005) and found that both heterozygous [−61.5%; (5.02 × 10^–4^ ± 2.73 × 10^–4^) cells/μm^2^] and homozygous [−79.1%; (2.73 × 10^–4^ ± 4.90 × 10^–5^) cells/μm^2^] mice had significantly reduced numbers of *EGFR*^+^ cells compared to control mice [(1.31 × 10^–3^ ± 2.09 × 10^–4^) cells/μm^2^] (Figures 4H–J,Q).

Neural stem/progenitor cells in the dLGE are highly proliferative and can form NS *in vitro*. To examine the properties of *Jag1* depletion in culture, cells from the dLGE were dissociated and cultured in the presence of EGF and evaluated for their propensity to form NS. The number of NS formed is an indicator of the number of actively dividing neural stem/progenitor cells. In our previous study, deletion of *Jag1* in homozygous mutants showed fewer numbers of NS (Blackwood, 2019); however, whether this phenotype is recapitulated in heterozygous mutants was not performed. We therefore, re-examined the loss of *Jag1* on the formation of primary NS. We found that the number of NS generated from the heterozygous [−44%; (12.2 ± 0.6) spheres] and homozygous [−69%; (6.7 ± 0.8) spheres] mice were significantly reduced relative to control [(21.9 ± 0.5) spheres] (Figures 4K–M,R). Moreover, the number of NS showed a substantial difference between the mutants (Figure 4R). These findings suggest that *Jag1* is required for the maintenance of stem-like cells *in vitro*, confirming previous findings that *Jag1* is required for the maintenance of stem cells *in vivo* (Nyfeler et al., 2005; Blackwood, 2019).

We next established that the reduced number of *EGFR*^+^ cells was not because of an increase in the programmed cell death. We performed a number of controls to address potential alternative explanations for the reduction of *EGFR*^+^ cells. First, we asked whether or not programmed cell death could contribute to the reduction of these cells. Using TUNEL labeling, we found no significant differences in the number of apoptotic cells in heterozygous [(3.37 × 10^–6^ ± 1.69 × 10^–6^) cells/μm^2^] or homozygous [(2.69 × 10^–6^ ± 1.90 × 10^–6^) cells/μm^2^] mice relative to controls [(5.38 × 10^–6^ ± 1.10 × 10^–6^) cells/μm^2^] (Figure 4S). To rule out the possibility that the *Foxg1*^*Cre*^ driver could contribute to our *in vitro* findings; we found no significant changes in sphere formation between the *Foxg1*^*Cre/+*^ [(22.33 ± 0.91) spheres] and *Foxg1*^+/+^ [(23 ± 0.73) spheres] mice (Supplementary Figures S2A–C). Altogether, these finding demonstrate that haploinsufficient *Jag1* leads to reduce neuronal division in the dLGE.

### Haploinsufficient *Jag1* Mice Show Reduced Neuronal Division in the RMS

Figure 5A shows a cartoon schematic of the RMS. Postnatally *Jag1* is expressed in the RMS (Stump et al., 2002; Nyfeler et al., 2005). Therefore, we examined whether the loss of *Jag1* affects neuronal cell division in the embryonic RMS. First, we determined whether *Jag1* is expressed in the RMS at E17.5. To this end, using double-labeled *in situ* hybridization, we tested whether probes for *Jag1* and *doublecortin* (*DCX*), a marker for migrating neuroblasts in the RMS (Gleeson et al., 1999) are coexpressed. Indeed, *Jag1* signal overlaps with *DCX* in the RMS (Figures 5B–D). When using an anti-PCNA antibody to examine proliferation, we found a substantial decrease in PCNA^+^ cells in heterozygous [−41.8%; (5.97 × 10^–3^ ± 2.21 × 10^–4^) cells/μm^2^] and homozygous [−65.9%; (3.50 × 10^–3^ ± 2.01 × 10^–4^) cells/μm^2^] mice relative to controls [(1.03 × 10^–2^ ± 1.49 × 10^–4^) cells/μm^2^] (Figures 5E–G,N). Similarly, Ki67 expression was also decreased in the same groups {*h* = [−51%; (3.54 × 10^–4^ ± 1.21 × 10^–5^) cells/μm^2^], *m* = [−70%; (2.13 × 10^–4^ ± 2.59 × 10^–5^) cells/μm^2^]} relative to control [(7.23 × 10^–4^ ± 4.54 × 10^–5^) cells/μm^2^] mice (Figures 5H–J,O). These findings suggest that the loss of *Jag1* appears to reduce cell division in the RMS. These observations of decreased cell division prompted us to ask whether progenitors in the RMS were specifically affected. We found that the expression of *Mash1*, a marker that labels the progenitor population (Guillemot and Joyner, 1993), was significantly reduced in heterozygous [−69.6%; (2.43 × 10^–3^ ± 7.54 × 10^–5^) cells/μm^2^] and homozygous [−75.9%; (1.90 × 10^–3^ ± 1.24 × 10^–4^) cells/μm^2^] mice compared to control [(7.97 × 10^–3^ ± 1.20 × 10^–4^) cells/μm^2^] mice (Figures 5K–M,P). The quantification of PCNA, Ki67, and *Mash1* (Figures 5N–P, respectively) also revealed significant differences between the heterozygous and homozygous mutant mice. Lastly, we examined whether the loss of progenitors could be due to an increase in cell death. To this end, no significant differences were found in the number of apoptotic cells in the heterozygous [(6.46 × 10^–6^ ± 2.01 × 10^–6^) cells/μm^2^] and homozygous [(5.92 × 10^–6^ ± 2.15 × 10^–6^) cells/μm^2^] mice compared to control [(7.00 × 10^–6^ ± 1.37 × 10^–6^) cells/μm^2^] (Figure 5Q) compared to controls.

### Haploinsufficient *Jag1* Displays Defects in the Generation of Periglomerular Interneurons

Neurons in the LGE migrate through the RMS to give rise to periglomerular cells in the OB (Figure 6A; Wichterle et al., 2001). The reduced cell division in the dLGE (Figure 4) and RMS (Figure 5) directed us to test whether reduced *Jag1* affects production of OB interneurons. First, we tested whether migrating neurons outside the mitral layer of the OB showed any defects in cell division. Using a PCNA antibody, significant decreases in PCNA^+^ cells were found in the heterozygous [−59.0%; (4.12 × 10^–4^ ± 1.01 × 10^–5^) cells/μm^2^] and homozygous [−84.9%; (1.51 × 10^–4^ ± 1.24 × 10^–5^) cells/μm^2^] mice compared to control [(1.01 × 10^–3^ ± 9.84 × 10^–5^) cells/μm^2^] mice (Figures 6B–D,K). Next, we examined whether mature interneurons were disrupted in the OB. *Tyrosine hydroxylase* (*TH*) and *glutamate receptor* 1 (*GluR1*) label major interneurons in the periglomerular layer (Kosaka et al., 1995; Montague and Greer, 1999). We observed significantly lower numbers of *TH*^+^ interneurons in the periglomerular layer of heterozygous [−46.8%, (2.92 × 10^–3^ ± 1.04 × 10^–4^) cells/μm^2^] and homozygous [−76.8%; (1.26 × 10^–3^ ± 1.22 × 10^–4^) cells/μm^2^] mice relative to control mice [(5.49 × 10^–3^ ± 1.75 × 10^–4^) cells/μm^2^] (Figures 6E–G,L; PG). Additionally, we quantified the number of *TH*^+^ interneurons that appear to be migrating (Figure 6L; mn) to the periglomerular layer. These were also decreased in the heterozygous [−60.0%; (9.58 × 10^–4^ ± 1.51 × 10^–4^) cells/μm^2^] and homozygous [−82.2%; (4.39 × 10^–4^ ± 1.02 × 10^–4^) cells/μm^2^] mutants compared to control [(2.41 × 10**^–^**^3^ ± 3.84 × 10**^–^**^4^) cells/μm^2^] (Figures 6E–G,L; mn). Similarly, *GluR1*^+^ interneurons within the periglomerular layer were decreased in heterozygous [−28.0%; (3.02 × 10**^–^**^3^ ± 6.0 × 10**^–^**^5^) cells/μm^2^] and homozygous mice (−61.0%; (1.63 × 10**^–^**^3^ ± 1.94 × 10**^–^**^4^) cells/μm^2^] relative to control [(4.20 × 10**^–^**^3^ ± 1.46 × 10**^–^**^4^) cells/μm^2^] mice (Figures 6H–J,M; PG). The quantification of PCNA, *TH*, and *GluR1* (Figures 6K–M, respectively) also showed significant decreases between the heterozygous and homozygous mutants. These results further suggest that while reduction in *TH*^+^ and *GluR1*^+^ interneurons could solely be due to proliferation defects, reduction in interneurons could also be explained by a relatively greater differentiation along with proliferation deficits in *Jag1* mutants.

The loss of *Jag1* causes premature neuronal differentiation in NS derived from the dLGE (Blackwood, 2019). Next, we asked whether there were any imbalances in differentiation of *TH* or *GluR1* interneurons and proliferation in the OB. To address this we quantified the relative ratio of differentiated interneurons vs. proliferating cells in the OB. The calculation of the migrating *TH*^+^ interneurons (mn) vs. PCNA cells ratio displayed no substantial differences in the heterozygous (0.976 ± 0.133) and homozygous (1.22 ± 0.322) mice compared to control (1.063 ± 0.234) (Figure 6N). Similarly, the ratio of periglomerular *TH*^+^ interneurons (PG) vs. PCNA cells also shows no significant changes in the heterozygous (1.321 ± 0.052) and homozygous (1.629 ± 0.275) mice in comparison to control (1.045 ± 0.119) mice (Figure 6O). However, we found that the ratio of *GluR1*^+^ interneurons (PG) vs. PCNA cells shows significant increases in the heterozygous (1.761 ± 0.061) and homozygous (2.853 ± 0.594) mutants compared to control (1.061 ± 0.079) (Figure 6P), suggesting an enhanced differentiation involving a subset of interneurons in the periglomerular layer.

Another possible explanation for the observed decrease in periglomerular interneurons is an increase in cell death. To this end, using TUNEL staining, we detected no significant changes in cell death between the heterozygous [(1.14 × 10**^–^**^5^ ± 6.73 × 10**^–^**^7^) cells/μm^2^] and homozygous [(1.21 × 10**^–^**^5^ ± 2.58 × 10**^–^**^6^) cells/μm^2^] mice compared with control [(1.08 × 10**^–^**^5^ ± 1.55 × 10**^–^**^6^) cells/μm^2^] mice (Supplementary Figures S3A–D). These observations suggest that loss of *Jag1* leads to defects in periglomerular interneurons due to the reduction in cellular proliferation and to premature differentiation, without significantly affecting cellular survival.

## Discussion

**FIGURE 7:**
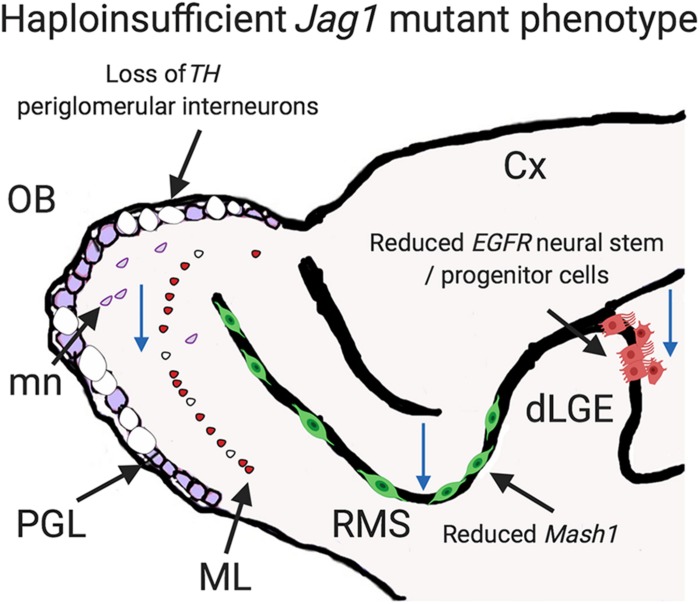
Schematic depicting how haploinsufficient phenotype of *Jag1* could disrupt the olfactory system at late embryonic development. *Jag1* is essential for the maintenance *EGFR*^+^ (large red) neural stem/progenitor cells in the dLGE. It is also critical for sustaining *Mash1*^+^ progenitors (green) in the RMS. Moreover, *Jag1* promotes a proliferative environment in the dLGE, RMS, and OB (blue arrows depict reduced cell division regions). In addition, loss of *Jag1* decreases the numbers of *TH*^+^ and *GluR1*^+^ bulbar interneurons in the periglomerular (PGL; blue and empty circles) and mitral layer (ML; small red). *TH*^+^ migrating neurons (mn; purple) are also reduced. Cx, cortex, dLGE, dorsal lateral ganglionic eminence; OB, olfactory bulb; mn, migrating neurons; PGL, periglomerular layer; ML, mitral layer; RMS, rostral migratory stream; dLGE, dorsal lateral ganglionic eminence; Cx, cortex.

This study provides new insights, through the examination of dose-dependent *Jag1*, into the regulation of Notch signaling. *Jag1* haploinsufficiency led to reduced overall LGE size, decreased cell division, and fewer numbers of neural stem/progenitor cells. This in turn was associated with reduced number of periglomerular interneurons (Figure 7).

Haploinsufficient JAG1 in humans causes Alagille syndrome (Li et al., 1997; Oda et al., 1997). Many of the features associated with Alagille syndrome have been recapitulated in heterozygous *Jag1* mice (Thakurdas et al., 2016), but double heterozygous *Jag1* and *Notch2* mice more closely approximated the phenotypes seen in humans (McCright et al., 2002). However, how the reduced *Jag1* levels affect the embryonic brain development was not previously explored. A prior study (Nyfeler et al., 2005) used mice doubly heterozygous for loss-of-function *Jag1* and *Notch1* alleles to examine cell division in the postnatal and adult SVZ. Our overall results regarding the impact of *Jag1* on cell division are consistent with their findings using double heterozygous mice. However, Nyfeler et al. (2005) did not find any effects of heterozygous mutants of *Jag1* or *Notch1* alone on cell division within the postnatal SVZ. One possible explanation for the discrepancy between their results and ours is that neuronal precursors that give rise to bulbar interneurons are particularly sensitive to the levels of *Jag1* in the embryonic stem cell niche. For example, studies have suggested that different types of interneurons are produced at various stages of embryonic and postnatal development (Batista-Brito et al., 2008), indicating potential differences in *Jag1’s* function at different ages. Future studies that examine proliferation at early stages of development in conditional Jag1 mutants may shed light on whether proliferation is specific to late development, as observed in our studies, or a general defect. These studies may clarify the differences with other mouse models where cell proliferation does not seem to be affected. Another possibility would be that *Jag1* haploinsufficient phenotypes during embryonic development were simply normalized due to compensatory changes occurring between embryonic and postnatal development. In addition, differing genetic backgrounds have been shown to modulate *Jag1* haploinsufficiency (Kiernan et al., 2007), and this could potentially contribute to differences between the studies.

*Jag1* is required to sustain *Sox2*-expressing stem/progenitor cells in the embryonic (Kiernan et al., 2006; Neves et al., 2011) and adult (Oesterle et al., 2008) inner ear. We observed that the expression of *Jag1* co-localized with a subset of *Sox2*-expressing neural stem/progenitor cells in the dLGE. Moreover, in the dLGE, reduced levels of *Jag1* failed to maintain an appropriate number of *EGFR*-expressing neural stem/progenitor cells and NS (Figure 4). These findings suggest that *Jag1* is involved in the maintenance of neural stem/progenitor cells in the dLGE. Relevant to this discussion, the expression of *Jag1* is also localized in the deep SVZ (Figure 2), a region that is known to be a source of adult stem cells that contributes to the generation of mature olfactory neurons (Young et al., 2007). Additionally, this deep expression is in proximity to ventral LGE progenitors that give rise to striatal projection neurons (Stenman et al., 2003). These observations suggest that Jag1 may function in various neural stem/progenitor pools giving rise to diverse neuronal cell types.

Lateral ganglionic eminence is comprised of *Gsh*-, *Dlx1/2-*, and *Mash1*-expressing progenitors that give rise to olfactory interneurons (Stenman et al., 2003; Batista-Brito et al., 2008). Mutations in *Gsh* (Toresson and Campbell, 2001; Yun et al., 2003) or *Dlx1/2* (Anderson et al., 1997; Bulfone et al., 1998) genes lead to the failure of OB interneuron development. In the present study, when visualizing the expression of *Dlx2* progenitors we found a reduced LGE size accompanied by fewer numbers of *TH-* and *GluR1*-expressing bulbar interneurons in *Jag1* mutant mice. These findings suggest that *Jag1* is essential for the development of the LGE and for maintaining the appropriate number of bulbar interneurons. It is relevant to note that the size of LGE in homozygous mice was significantly increased in comparison to heterozygous mice. On the other hand, we found that cell division, neurosphere formation, and the number of *Mash1*-expressing progenitors in the homozygous mice were significantly decreased when compared to heterozygous mice. These apparently paradoxical findings could either point toward a role of Jag1 relevant to the maintenance of the size of LGE that has not been investigated, or to a dose-dependent increase in cellular differentiation from heterozygous to homozygous mutants.

Notch signaling is involved in cell fate decisions (Weinmaster, 1997; Artavanis-Tsakonas et al., 1999). In the context of cell fate, we propose two models for *Jag1*. In the first model, Jag1 stimulates progenitor’s identity through self-renewability, thereby maintaining a pool of progenitors to give rise to bulbar interneurons. This is consistent with our previous study that showed that reduced levels of *Jag1* showed dramatic reduction in the number of self-renewing NS (Blackwood, 2019). Moreover, this model is supported by previous reports that demonstrated *Jag1* is required for the self-renewal of NSCs in the postnatal SVZ (Nyfeler et al., 2005) and the adult dentate gyrus (Lavado and Oliver, 2014). Further supporting this model are findings that inhibition of Notch signaling reduces neural stem cell’s self-renewability (Imayoshi et al., 2010) and diminishes radial glial identity (Gaiano et al., 2000), suggesting that activation of Notch is vital for the maintenance of NSCs. Lastly, our findings that *Jag1* mutants had significant reductions in the numbers of NS in culture and in *EGFR*-expressing neural stem/progenitor cells with self-renewal abilities are consistent with this model.

In the second model, Jag1 is required to prevent the depletion of the neural stem/progenitor pools by inhibiting premature differentiation. This model is supported by our current findings that *Jag1* mutants showed an enhanced differentiation in the *GluR1*-expressing interneurons (Figure 6). Moreover, in our previous studies, the observations that *Jag1* deficient mice showed precocious differentiation of cortical *Tbr2*-expressing intermediate progenitors and premature differentiation of NS are further evidences implicating Jag1 role in neuronal differentiation (Blackwood, 2019). These findings are consistent with a previous study that showed that Jag1 inhibits differentiation of adults NSCs (Ottone et al., 2014). How Jag1 inhibits neuronal differentiation in the LGE is unclear. The promoter of *Jag1* is bound and regulated by *FoxP1*, transcription factor (Braccioli et al., 2017). *Foxp1* has been shown to promote differentiation of NSC toward neuronal lineage (Araujo et al., 2015; Precious et al., 2016; Braccioli et al., 2017). Interestingly, it was shown that *FoxP1* is not only required for neuronal differentiation, but also functions to repress the expression levels of *Jag1* (Braccioli et al., 2017), suggesting that once matured, neuronal cells downregulate Jag1 signaling. Overall, these findings support a model in which Jag1 maintains neural stem/progenitor pools by inhibiting their premature neuronal differentiation.

Finally, we note that homozygous loss of Jag1 does not completely eliminate periglomerular interneurons in the bulb. This suggests the possibility of alternative means of generating mature interneurons that are not dependent on Jag1 signaling. Future studies in the expression of the remaining Notch ligands (e.g. Jag2) or the presence of Notch-independent signaling modules within the dLGE might explain this observation.

## Data Availability Statement

The raw data supporting the conclusions of this article will be made available by the authors, without undue reservation, to any qualified researcher.

## Ethics Statement

The animal study and animal protocols were reviewed and approved by the Cornell University’s Institutional Animal Care and Use Committee (IACUC).

## Author Contributions

CB performed all the experiments and designed the experiments. AB performed the western blot. TG provided the *Jag*^*fl/fl*^ mouse. CB, SN, and JH prepared the manuscript.

## Conflict of Interest

The authors declare that the research was conducted in the absence of any commercial or financial relationships that could be construed as a potential conflict of interest.
