# Supplementary material for: Notch Dosage: Jagged1 Haploinsufficiency Is Associated With Reduced Neuronal Division and Disruption of Periglomerular Interneurons in Mice
Source: Front Cell Dev Biol. 2020 Feb 26;8:113. doi: 10.3389/fcell.2020.00113 (PMC7054221; doi:10.3389/fcell.2020.00113)
Supplement: Supplementary file 1 [file Data_Sheet_1.PDF]

Supplementary Material

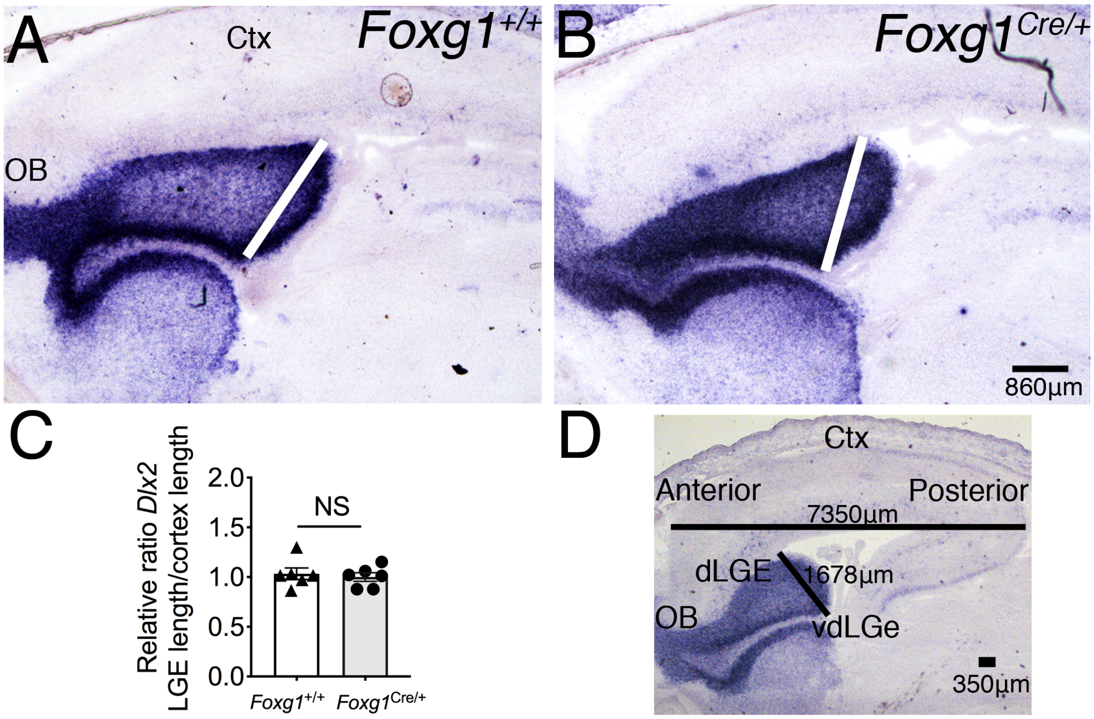

Supplemental Figure 1. *Foxg1* mice show no defects in the size of the LGE.
(A, B) *In situ* hybridization expression of *Dlx2* mRNA probes on murine 20µm sagittal brain sections at embryonic day 18.5. **(C)** Quantification of the relative ratio of the LGE to cortex in the $Foxg1^{+/+}$ (0.296 ± 0.013) and $Foxg1^{Cre/+}$ (0.306 ± 0.017) mice showed no significant differences (p = 0.145). Scale bar=860µm **(D)** Embryonic day 17.5 brain section labeled with *Dlx2* mRNA probe. Example of the segments used to calculate the length of the LGE, which is represented as a ratio of LGE to cortex. The length of the LGE is defined by drawing a line from the dLGE to the vLGE (short back line). The length of the cortex is defined by drawing a straight line from the apex of the posterior cortex to the anterior prefrontal cortex (long black line). Scale bar=300µm. 1 section from each of the 5 independent embryos (n=5) was counted for each genotype. Key to statistics: NS, not significant. Graph shows the means ± SEM and normalized to $Foxg1^{+/+}$ mouse. Stats were performed using unpaired two-tailed Student's t-test. Ctx, Cortex; dLGE, dorsal lateral ganglionic eminence; vLGE, ventral lateral ganglionic eminence; OB, olfactory bulb.

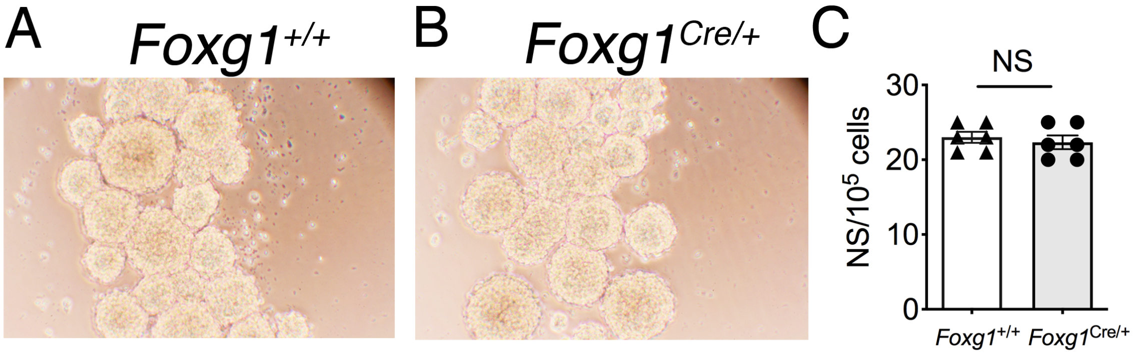

Supplemental Figure 2. Primary neurospheres isolated from the *Foxg1* mice show no changes. Embryonic day 17.5 primary neurospheres after 7 days *in vitro* isolated from the dLGE of **(A)** *Foxg1*$^{+/+}$ (23.00 ± 0.73 spheres) and **(B)** *Foxg1*$^{Cre/+}$ (22.33 ± 0.91 spheres) mice show no significant ($p = 0.5826$) changes. **(C)** Quantification of the average number of primary neurospheres per field of view. Key to statistics: NS, not significant. 1-2 pictures from 5 independent embryos (n=6) were counted for each genotype. Graph shows the means ± SEM. Stats was performed using unpaired two-tailed Student's t-test.

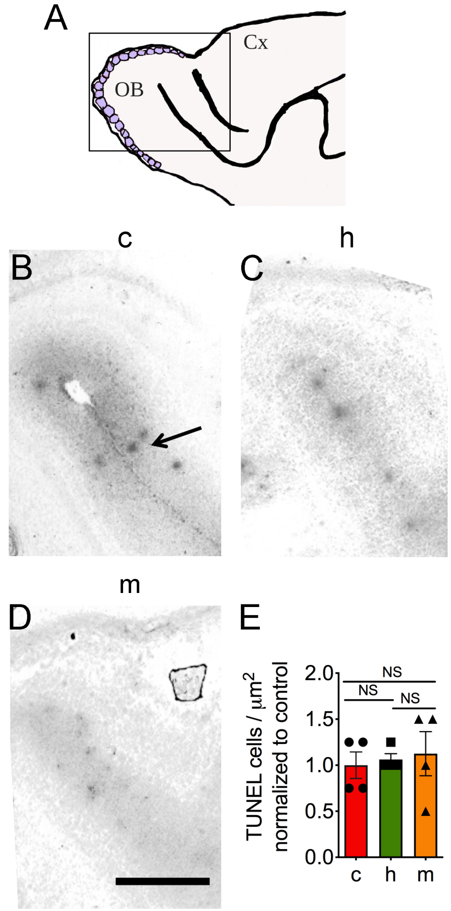

Supplemental Figure 3. Loss of *Jag1* shows no changes in apoptotic cells in the olfactory bulb. (A) Schematic cartoon shows a sagittal region of the olfactory bulb. TUNEL staining on E17.5 sagittal brain sections from **(B)** control (c) ((1.08 $x10^{-5}$ ± 1.55 $x10^{-6}$) cells / μm^2), **(C)** heterozygous (h) ((1.14 $x10^{-5}$ ± 6.73 $x10^{-7}$) cells / μm^2), and **(D)** homozygous (m) ((1.21 $x10^{-5}$ ± 2.58 $x10^{-6}$) cells / μm^2) mice show no substantial changes ($p = 0.8688$) in cell death. **(E)** Similarly, quantification of the total number of apoptotic cells per olfactory bulb shows no significant changes (c, n=4; h, n=4; m, n=4). Scale bar=200μm. Graph shows the means ± SEM. Key to statistics: NS, not significant. 1-2 pictures from 3 independent embryos (n=3) were counted for each genotype. Stat was performed using one-way ANOVA.
